# Dust in and Around the Heliosphere and Astrospheres

**DOI:** 10.1007/s11214-022-00939-7

**Published:** 2022-12-06

**Authors:** Veerle J. Sterken, Lennart R. Baalmann, Bruce T. Draine, Egor Godenko, Konstantin Herbst, Hsiang-Wen Hsu, Silvan Hunziker, Vladislav Izmodenov, Rosine Lallement, Jonathan D. Slavin

**Affiliations:** 1 Institute for Particle and Astroparticle Physics, ETH Zürich, Zürich, Switzerland; 2 Dept. of Astrophysical Sciences, Princeton University, Princeton, NJ USA; 3 Moscow Center for Fundamental and Applied Mathematics, Lomonosov Moscow State University, Moscow, Russia; 4 Space Research Institute of Russian Academy of Sciences, Moscow, Russia; 5 Institut für Experimentelle and Angewandte Physik, Christian-Albrechts-Universität zu Kiel, Kiel, Germany; 6 Laboratory for Astrophysics and Space Planetary, University of Colorado, Boulder, USA; 7 GEPI, Observatoire de Paris, Meudon, France; 8 Center for Astrophysics | Harvard & Smithsonian, Cambridge, MA USA

**Keywords:** Cosmic dust, ISM, LIC, Interstellar dust, Heliosphere, Astrosphere

## Abstract

Interstellar dust particles were discovered in situ, in the solar system, with the *Ulysses* mission’s dust detector in 1992. Ever since, more interstellar dust particles have been measured inside the solar system by various missions, providing insight into not only the composition of such far-away visitors, but also in their dynamics and interaction with the heliosphere. The dynamics of interstellar (and interplanetary) dust in the solar/stellar systems depend on the dust properties and also on the space environment, in particular on the heliospheric/astrospheric plasma, and the embedded time-variable magnetic fields, via Lorentz forces. Also, solar radiation pressure filters out dust particles depending on their composition. Charge exchanges between the dust and the ambient plasma occur, and pick-up ions can be created. The role of the dust for the physics of the heliosphere and astrospheres is fairly unexplored, but an important and a rapidly growing topic of investigation. This review paper gives an overview of dust processes in heliospheric and astrospheric environments, with its resulting dynamics and consequences. It discusses theoretical modeling, and reviews in situ measurements and remote sensing of dust in and near our heliosphere and astrospheres, with the latter being a newly emerging field of science. Finally, it summarizes the open questions in the field.

## Introduction

The heliosphere plows through the local interstellar medium (ISM) at a velocity of about $26\mbox{ km}\,\mbox{s}^{-1}$ (Witte et al. 1993; Lallement and Bertaux 2014). As a consequence, neutral interstellar gas and dust particles move through the heliosphere and can be measured in situ, presenting a unique opportunity to enhance our knowledge of the heliosphere as a proxy for other astrospheres, including the dust component.

The first predictions and studies of the dynamics of interstellar dust (ISD) passing through the heliosphere were made in the 1970s (Bertaux and Blamont 1976; Levy and Jokipii 1976; Gustafson and Misconi 1979; Morfill and Grün 1979). The ISD was directly detected in situ for the first time by the *Ulysses* dust detector system (Grün et al. 1993), providing insights into the dust size distribution, its flow direction, and in particular its dynamics in the heliosphere. The first few candidate samples of contemporary ISD were brought back to the Earth by the *Stardust* mission for laboratory investigation (Westphal et al. 2014).

Similar to the heliosphere, astrospheres of other stars also plow through their immediate interstellar environments. These systems have a large variety of dust, gas, and stellar properties. Just like the heliosphere is a proxy for other astrospheres, other astrospheres can be proxies for studying the history and future of our heliosphere as it journeys through very different interstellar medium environments.

While remote observations of dust interactions with astrospheric structures are now possible, *in situ* measurements of dust in astrospheres will always be problematic. Therefore, the in situ study of dust in the heliosphere presents a unique opportunity to explore, model, and understand the interaction of interstellar (and interplanetary) dust particles with other stellar astrospheres. A brief overview of the history of heliospheric ISD modeling and in situ detection is given in Zank et al. (2022, this volume).

Interstellar dust plays an essential role in the interstellar medium: for astrochemistry (as sites for creating complex molecules), for shielding of UV light in molecular clouds such that molecules can survive, as an agent for removing free electrons (and ions) from the gas, and for the temperature regulation of interstellar clouds. In addition, dust is a constituent (besides the gas) that forms the protoplanetary disk around newborn stars from which planets (and ultimately life) are ‘born’. The ISD is also crucial for astronomical observations since it is the foreground through which astronomers observe. Its properties are often used for modeling protoplanetary disks to aid observational interpretations. Our knowledge of the composition and size distribution of ISD mostly results from models based on astronomical observations, and direct measurements are needed to validate these models. For all these reasons, detecting the local ISD *in situ* and constraining its properties is very important. An in-depth review of the ISD in the local interstellar cloud (LIC) and in the solar system is given by Sterken et al. (2019).

Apart from its role in astrophysics, *in situ* ISD research is vital for the interdisciplinary science case of the interaction of the dust with the heliosphere/astrosphere. The dust particles are charged; the tiniest of these particles (nanodust) have the highest charge-to-mass ratios and thus couple closely to the heliospheric magnetic field on small (≪ 1 AU) scales. Mid-sized (sub-μm-sized) dust couples to the magnetic field on larger scales but is still influenced by Lorentz forces. Since the heliospheric magnetic field varies over the 22-year magnetic solar cycle, the dust can be used to indirectly probe heliospheric properties (e.g., its dynamic structure), providing an extra constraint for heliosphere models. Dust may also play an important role in the physics of the heliosphere/astrospheres that is so far very little explored. The state-of-the-art of dust-heliosphere science, and current open science questions concerning dust-heliosphere interactions, are summarized in Sterken et al. (2022).

This review paper covers theoretical modeling and measurements, remote and in situ, of interstellar dust near and in the heliosphere and around stellar astrospheres, and their interaction with the local plasma/gas. We start by describing the physical processes between the dust and the heliosphere, including dust charging in different regions of the heliosphere, sputtering, collisions, sublimation, and centrifugal disruption (see Sect. 2). Section 3 discusses the dust dynamics in the heliosphere, related to some of these processes. In particular, we discuss the influence of the solar cycle on the dust fluxes and the pile-up and filtering of ISD near the heliospheric/astrospheric boundaries and the consequences for in situ measurements. Section 4 discusses current models of ISD dynamics in the heliosphere. Three types of models are described: Monte Carlo trajectory simulation models, magnetohydrodynamic+kinetic models, and Lagrangian fluid-based models. Section 5 describes in situ measurements of dust in the heliosphere. Remote sensing measurements of dust near the heliosphere are described in Sect. 6. Section 7 sheds light on the newly emerging field of remote sensing measurements of dust near and in astrospheres, and Sect. 8 summarizes the most important open science questions.

## Physical Processes of Dust in and Around the Heliosphere

To address physical processes shaping the dynamical evolution of sub-micron to μm-sized grains from interstellar space to the heliosphere, we focus these discussions on the grain-plasma interactions, including charging, sputtering, collisions, sublimation and centrifugal disruptions.

### Grain Charging

The grain charge is determined by various charging currents that result from exposure of the dust to the ambient plasma and ionizing ultraviolet UV radiation (Horanyi 1996). The electric charge on dust grains directly determines the charge-to-mass ratio of dust grains ($q/m$) and thus the influence of electromagnetic forces on their dynamical evolution. To first order,^1^ dust grains are charged to the same electric potential determined by the ambient plasma/radiation conditions.

For dust grains with radius $a$, the amount of electric charge ($Q_{ \mathrm{d}}$) is proportional to the grain size and the surface electric potential ($\phi $ in Volt) and can be written as:

1 $$ Q_{\mathrm{d}} = 4\pi \epsilon _{0}\, a\, \phi \;, $$

where $\epsilon _{0} = 8.8541\cdot 10^{-12}$ A^2^ s^4^ kg^−1^ m^−3^ is the vacuum permittivity. Furthermore, the grain charge-to-mass ratio increases with decreasing grain radii as $q/m \propto \phi \, a^{-2}$, indicating that grain charging and the effects of the Lorentz force are more important for smaller grains.

The temporal evolution of grain charge can be calculated by including charging currents from electron and ion collection ($J_{\mathrm{e}}$ and $J_{\mathrm{i}}$), photoemission ($J_{\mathrm{ph}}$), and secondary electron emission ($J_{\mathrm{sec}}$), and be written as:

2 $$ \frac{\mathrm{d}Q_{\mathrm{d}}}{\mathrm{d}t} = J_{\mathrm{e}}+J_{ \mathrm{i}}+J_{\mathrm{ph}}+J_{\mathrm{sec}}\;. $$

Under normal solar system conditions, the most important charging current is due to ambient plasma electrons ($J_{\mathrm{e}}$). Assuming ion and electron temperatures are the same, the lighter electrons, because of their higher mobility, constitute a negative current with an amplitude higher than that of the positive ion current ($J_{\mathrm{i}}$) by a factor of $\sqrt{m_{\mathrm{i}}/m_{\mathrm{e}}} > 43$, where $m_{\mathrm{i}}$ and $m_{\mathrm{e}}$ are the ion and electron mass, respectively (Horanyi 1996; Spitzer 1941). If only plasma ion and electron collection are considered, there is a negative grain potential at equilibrium. For a Maxwellian proton-electron plasma, the equilibrium grain potential ($\phi _{\mathrm{eq}}$) can be solved analytically as $\phi _{\mathrm{eq}} \approx -2.5\, k\,T_{\mathrm{e}}/e$ (Spitzer 1941), where $k\,T_{\mathrm{e}}$ is the plasma electron temperature in eV and $e$ is the elementary charge.

Ionizing radiation, such as solar and stellar UV and energetic particles, produces photoelectrons ($J_{\mathrm{ph}}$) and secondary electrons ($J_{ \mathrm{sec}}$) and results in charging currents that counterbalance the electron collection current, leading to a more positive grain potential. A significant solar UV flux comes from the Lyman-$\alpha $ emission at a wavelength of 121.6 nm, at an energy of 10.2 eV that exceeds the typical work function^2^ (approximately 6–8 eV) of candidate ISD materials (see Fig. 1 of Draine 1978), allowing photoelectron production. Similarly, plasma electrons with sufficient energy will create secondary electrons, leading to similar charging effects (Meyer-Vernet 1982). In addition, the photoelectric yields for grains with sizes comparable to or smaller than the photon penetration depth (of order 10-50 nm) can be enhanced (Watson 1973; Draine 1978), leading to a more positive potential. Secondary electron yields can be similarly enhanced for grain sizes smaller than or comparable to the electron penetration length (of order 10s of nm for ∼ 100 eV electrons), also leading to more positive grain potential (Draine and Salpeter 1979b; Chow et al. 1993; Ma et al. 2013; Slavin et al. 2012). This effect also leads to higher charges for dust aggregates consisting of small particles (Ma et al. 2013). The secondary electron yield from energetic ions is generally much lower and thus less relevant for ISD charging.

While the steady-state grain potential is determined by plasma temperature, the charging time (i.e., the time for grains to reach $\phi _{\mathrm{eq}}$) largely depends on plasma density. For grains in low-density plasmas, the charging time could be long compared to their traverse time, meaning that the grain potential could deviate significantly from the local equilibrium potential. The same applies to very small grains (nanodust), since charging time increases with decreasing grain cross-section. In addition, considering the quantized nature of electric charges, the charging currents can no longer be treated as “continuous” under these conditions. Various numerical methods have been developed to model discrete grain charging (Draine and Sutin 1987; Cui and Goree 1994), which are overall more computationally expensive than the continuous current calculations.

### Surface Charges in and Near the Heliosphere

**Fig. 1:**
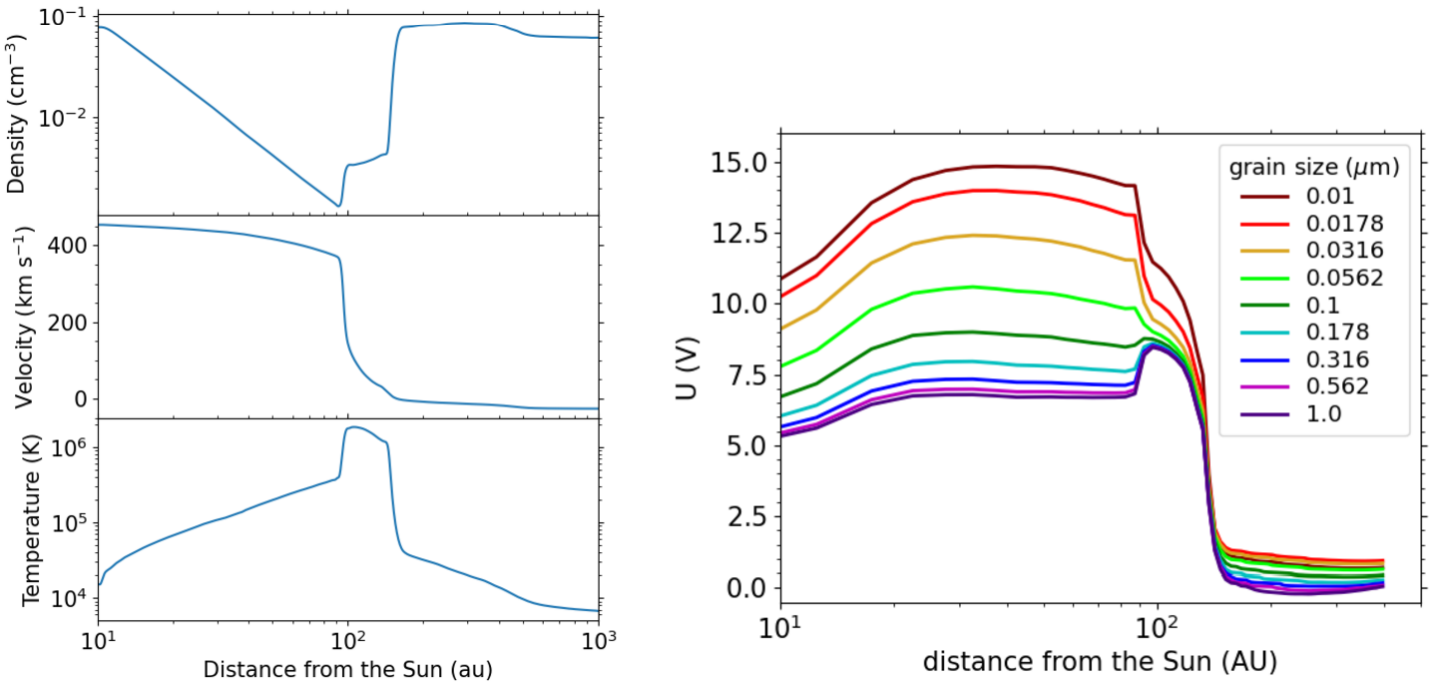
The heliosphere properties from MHD+kinetic modeling (left), including heating of the plasma by pickup ions and the corresponding (calculated) dust particle surface charge (right), with distance from the Sun through the different regions of the heliosphere, including the small particle effect, from Slavin et al. (2012), p. 2 $\copyright $ AAS. Reproduced with permission

**Fig. 2:**
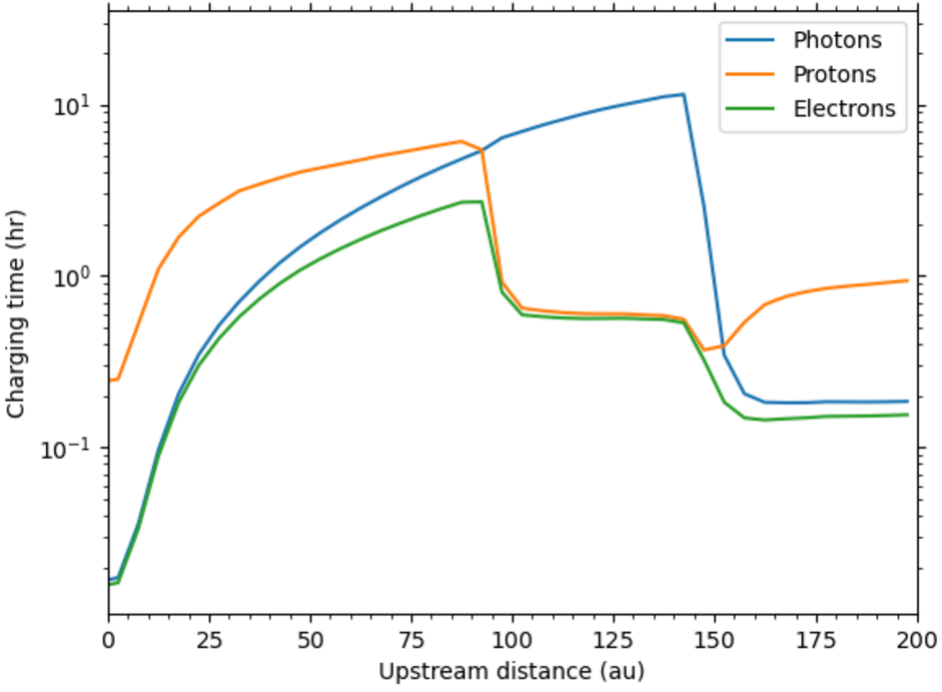
The charging time for dust grains with radius of 0.1 μm versus the distance to the Sun for the currents from photoionization, electrons, and ions from the plasma

The charging environment in the inner heliosphere is dominated by the Sun, i.e., the solar wind and solar UV radiation. Since the solar wind electron density and solar UV flux decrease outward with the inverse square of heliocentric distance, the grain equilibrium potential remains roughly constant over the inner heliosphere, at around $\phi _{eq} \cong +5$ to +15 V, depending on the secondary electron yield (Slavin et al. 2012) and plasma temperature. The potential also depends on the composition of the dust (e.g. Kimura and Mann 1998) and the morphology (Ma et al. 2013). In the heliosheath the plasma density is lower than in the VLISM and the temperatures are higher (see Fig. 1, left^3^), which leads to a higher grain potential for particles that are larger than 100 nm (Fig. 1, right, from Slavin et al. 2012). Dust grains outside of the heliosphere have a small electric potential of about −0.2 to +1 V depending on their size and composition, due to the low temperatures and weak UV field. Nanodust outside the heliosphere may pile up near the heliopause (Slavin et al. 2012) and can undergo acceleration effects due to stochastic charging (Hoang et al. 2012). Charging timescales are in the range of several hours for dust grains with radius of about 0.1 μm in the heliosheath, and on the order of minutes to hours in the ISM (see Fig. 2). The charging times are shorter for larger particles (Draine and Sutin 1987; Horanyi 1996). The charging times are short in comparison with the time for the dust particles to reach different plasma regions in the heliosphere.

### Sputtering

Another effect of ambient plasma is sputtering, an erosion process driven by incident ions. In the first order, the grain sputtering lifetime is proportional to grain size, i.e., the sputtering lifetime is shorter for smaller grains. However, considering the sputtering yield caused by solar wind protons of the order of 10^−2^ (Schmidt and Arends 1985), the sputtering lifetime of ISDs is of the order of 10^5^ years, much longer than their traversal time in the heliosphere. During the long residence times of ISD particles in the interstellar medium, particles that are overtaken by supernova blastwaves can undergo substantial erosion and even total destruction. The resulting grain lifetimes are uncertain, but recent estimates suggest ∼350 Myr as a reasonable estimate (Draine and Salpeter 1979a; Jones et al. 1994; Bocchio et al. 2014), implying that most solid material in the ISD population must have been grown in the interstellar medium (Draine 2009a). One recent grain model concludes that the bulk of the interstellar grain population consists of grains that individually contain multiple materials, including approximately equal volumes of amorphous silicates and hydrocarbons (Hensley and Draine 2022).

### Collisions

Grain-grain collisions are not a significant process for ISDs in the heliosphere. Assuming a homogenous interplanetary dust density of 50 km^−3^ with a characteristic grain size of 2 μm, the collisional probability of ISDs passing through the heliosphere is negligibly low at around 10^−5^. However, large zodiacal dust particles and meteoroids will have $\sim 10^{7}$ impacts of submicron ISD particles per cm^2^ per Myr of exposure.^4^ With ISD impact speeds of $\sim 30$ km/s, this will result in microcratering. The ISD particles will be vaporized, and the larger zodiacal dust particles will undergo gradual erosion of the surface. If the mass of material excavated from one microcrater is $\sim 10^{2}$ times the mass of the ISD impactor, this will result in an erosion rate of $\sim 0.3$ μm/Myr.

### Sublimation

Solar heating will only produce sublimation of refractory grain materials (e.g., silicates, metal oxides, hydrocarbons) if the grains approach very close (within $\sim 0.01$ AU) to the Sun. Ices, if present, could be sublimed at distances of $\sim 5$ AU, but the ISD population entering from the local diffuse ISM is thought to be ice-free, as H_2_O ice absorption features are absent in the diffuse ISM (e.g., Whittet et al. 1997). Sublimation therefore appears to be unimportant for ISD grains in the heliosphere except very close to the Sun.

### Centrifugal Disruption

Radiative torques on grains resulting from scattering and absorption of anisotropic starlight are understood to produce spin-up of grains in the diffuse ISM to appreciable rotational speeds (e.g., Draine and Weingartner 1996, 1997). Within the heliosphere, the much higher intensity of solar radiation will spin grains up to higher rotational velocities. ISD particles entering the Solar system may be disrupted if they approach within a few AU of the Sun (Silsbee and Draine 2016). If ISDs have low tensile strengths, this effect can be important at even larger distances (Hoang 2019).

## Dynamics of Dust in and Around the Heliosphere

This section briefly describes the dynamics of interstellar dust moving in and through the heliosphere. We start by describing the dynamics of the dust in the supersonic solar wind, followed by a discussion on the filtering effect this has on the dust flow, and finally, we discuss the filtering in the heliospheric boundary regions. An in-depth description of the ISD dynamics in the supersonic solar wind is given in Landgraf (2000), Sterken et al. (2012). The filtering effect is described in Landgraf et al. (2000), Sterken et al. (2013) and the filtering in the boundary regions of the heliosphere is discussed in detail in Kimura and Mann (1998, 1999), Linde and Gombosi (2000), Czechowski and Mann (2003b,a), Slavin et al. (2012), Alexashov et al. (2016).

Three forces dominate the dynamics of ISD in the heliosphere: solar gravity, solar radiation pressure, and Lorentz force. Their dominance depends on the size, charge and optical properties of a particle and on its location in the solar system (e.g., Fig. 1 in Sterken et al. 2012 from Landgraf 1998). Other forces may also be important for interplanetary dust particles (IDP), in particular the Poynting–Robertson drag and planetary perturbations, due to the longer residence times while being on elliptical (Keplerian) orbits. However, planetary perturbations, Poynting–Robertson drag, the Yarkowsky effect,^5^ and solar wind corpuscular drag can be neglected for ISD in our heliosphere (Altobelli 2004). Although IDPs and nanodust from the inner solar system may play an important (but so far not yet well explored) role in heliosphere physics, this paper focuses mainly on ISD.

The equation of motion for ISD particles can be formulated as follows:

3 $$ {\mathbf{\ddot{r}}} = - \frac{(1-\beta )GM_{\odot}}{|{\mathbf{r}}^{3}|}{\mathbf{r}} + \frac{Q}{m} \left ( {\dot{\mathbf{r}}}_{\mathrm{p},\mathrm{sw}} \times \mathbf{B_{sw} }\right ) $$

with **r** the position vector of the particle, $\beta $ the ratio of solar radiation pressure force and gravity, $G$ the gravitational constant, $M_{\odot}$ the mass of the Sun, $\frac{Q}{m}$ the charge to mass ratio of the dust particle, ${\dot{\mathbf{{r}}}}_{\mathrm{p},\mathrm{sw}}$ the velocity of the dust particle with respect to the solar wind, and ${\mathbf{B}}_{\mathrm{SW}}$ the solar wind magnetic field. The following sections describe the motion of ISD in the heliosphere, sorted by particle size.

### Dynamics of Micron-Sized ISD Moving Through the Heliosphere

**Fig. 3:**
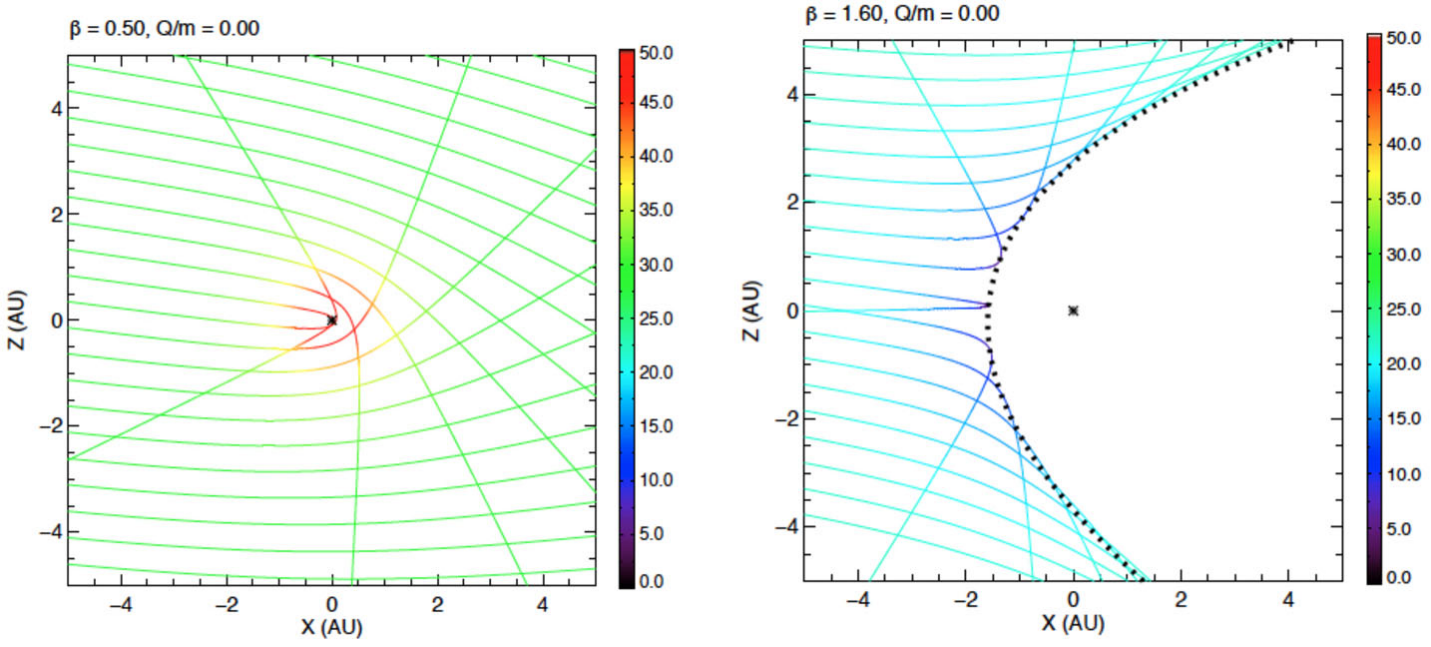
Trajectories of dust grains with $\beta =0.5$ (corresponding to 0.8 μm radius) and with $\beta = 1.6$ (corresponding to ca. 0.2 μm radius). A so-called $\beta $-cone is visible for $\beta $ larger than 1. Credit: Sterken et al. (2012), p. $3 - 4$, reproduced with permission $\copyright $ ESO

**Micron-sized ISD** that moves into the heliosphere is mainly affected by solar gravity. Particles larger than a micrometer have higher number densities downstream from the Sun, due to gravitational focusing (e.g., Fig. 3, left). While interplanetary dust revolves around the Sun for a thousand to a million years and the Poynting–Robertson drag and collisions dominate its long-term evolution,^6^ the ISD passes through the solar system in only about 50 years (about 5.5 AU per year on average).

The solar radiation pressure plays a significant role for particles smaller than one micrometer. The ratio of the solar radiation pressure and solar gravity ($\beta $) depends mainly on the particle size, composition, density, and surface morphology. For each particle with a fixed set of these properties (hence, not changing in time due to processes like sublimation, collision, etc.), a so-called $\beta $-curve relates the $\beta $-value of the particles to their size. $\beta $-curves can either be retrieved through experiments in the laboratory (e.g., Gustafson 1994) or by Mie calculations (e.g., Draine and Lee 1984). Each particle size has one $\beta $-value, but some $\beta $-values can have two corresponding particle sizes. The $\beta $-curve has a maximum value typically ranging from below one for certain silicates to five for darker materials like carbon or graphite (e.g., Kimura and Mann 1999). Examples of $\beta $-curves are given in a.o. Schwehm (1976), Gustafson (1994), Kimura and Mann (1999), Kimura et al. (2003), Silsbee and Draine (2016).

Trajectories of interstellar dust particles with $\beta = 1$ are straight lines everywhere in the solar system since the solar radiation pressure force cancels out the solar gravitational force. Particles with $\beta > 1$ are repelled from the Sun and a void region is formed downstream from the Sun, often referred to as the $\beta $-cone (see Fig. 3, right). The size of this conical region depends on the $\beta $-value of the particles and hence, can be used in conjunction with spacecraft measurements of the particle masses to constrain dust properties – through the dust dynamics – if sufficient statistics are available (e.g., Landgraf et al. 1999; Kimura et al. 2003). This procedure is sometimes referred to as $\beta $*-spectroscopy* (Altobelli 2004). Spacecraft close to the Sun can not capture or measure in situ particles in a mass range for which $\beta $ is larger than the $\beta $-value corresponding to the $\beta $-cone at that location. The closer to the Sun, the larger that ‘missing’ mass range is, also called the $\beta $*-gap*.

### Dynamics of Sub-Micron Sized Interstellar Dust Moving Through the Heliosphere

**Fig. 4:**
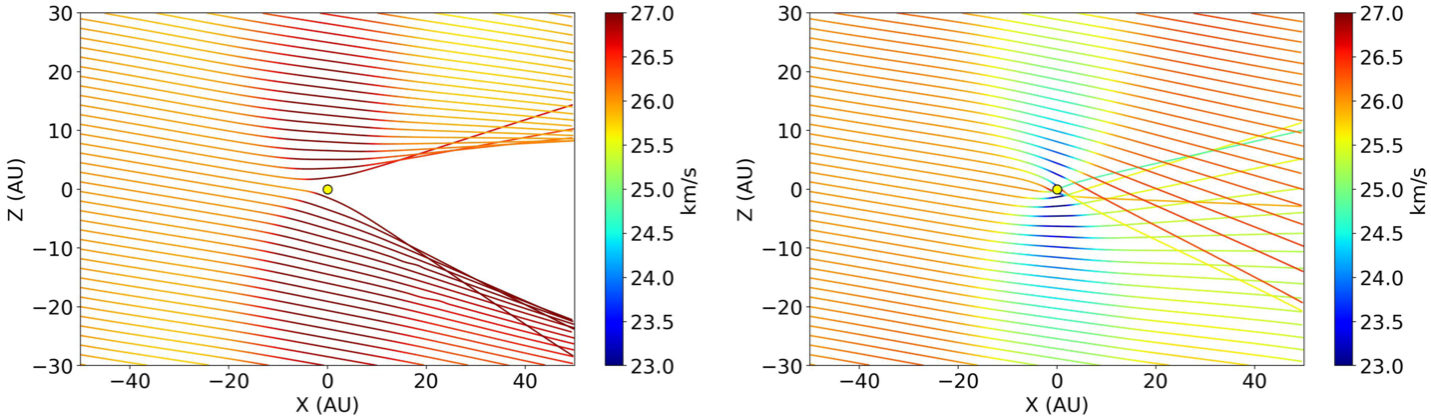
Trajectories of dust grains with Q/m = 0.5 C kg^−1^ (corresponding to ca. 0.4 μm radius) during the defocusing phase (start of simulation at 50 AU distance, in 2013) and during the focusing phase of the solar cycle (start of simulation at 50 AU distance, in 1999) with $\beta =1$ so only the effect of Lorentz force is illustrated in this example. The colors show the particle velocities in the heliocentric frame in km s^−1^

**Submicron-sized ISD** is, in addition to the solar gravity and radiation pressure forces, also subject to Lorentz forces when particles are smaller than about half a micrometer. The dust particles that move through a plasma and are subject to UV radiation acquire a net surface charge (see Sect. 2). While they move inward at (on average) about 26 km s^−1^, the solar wind velocity (400 km s^−1^ or more, depending on position and time in space) dominates the resulting Lorentz forces. In regions where the solar wind is slowed down, the particle velocity and solar wind velocity will play equally important roles. This is especially important in the outer heliosphere regions like the heliosheath. Since the azimuthal component of the solar wind magnetic field is predominant in a large part of the heliosphere (assuming a Parker spiral structure, see also Landgraf 2000; Sterken et al. 2012), the resulting Lorentz force is directed mainly to the north or south directions, with the directions depending on the polarity of the solar magnetic field and on the sign of the dust charge. The sign of the IMF depends on the solar cycle and on the position of the dust particle in the IMF. The net effect of the Lorentz force on ISD is a 22-year cycle where focusing and defocusing of ISD with respect to the solar equatorial plane alternates, as illustrated in Fig. 4. During solar minima, this effect is very strong. At solar maximum, large HCS tilt results in particles experiencing both inward and outward magnetic fields so the total effect is reduced.

The smaller the particles are, or the larger their surface area, e.g., for dust aggregates, the higher their charge-to-mass ratio is, and hence, the larger their susceptibility to the Lorentz force. Since the charge also depends on the dust composition, the heliosphere can metaphorically speaking be regarded as a giant mass spectrometer. The filtering effect of the heliosphere as a consequence of the dust dynamics is further discussed in Sect. 3.4.

**Fig. 5:**
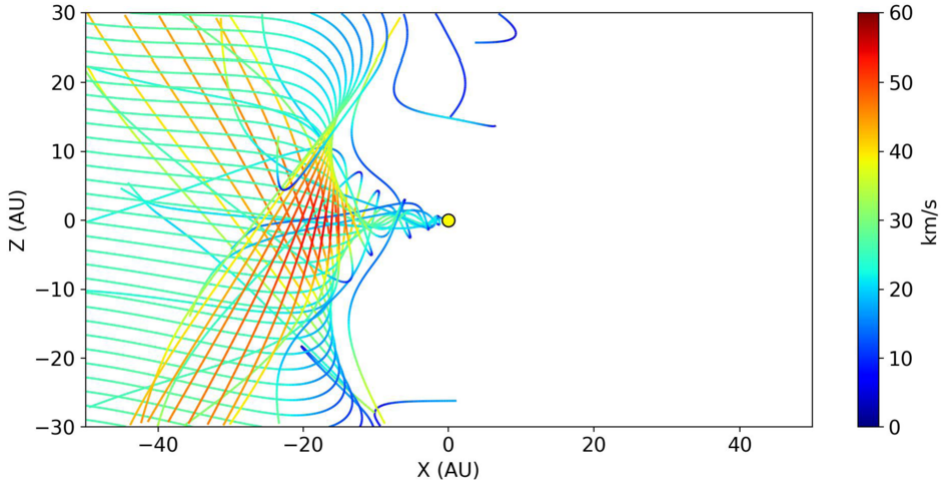
The flow pattern of smaller dust particles (about 12 nm in this case, ignoring the effects of the heliopause and the small particle effect) are much more complicated and less intuitive. This can lead to higher concentrations in the solar system at certain times and positions, also described as ‘waves’ of small dust ‘rolling’ into the heliosphere (e.g., Hunziker et al. 2022a; Sterken et al. 2022)

Higher charge-to-mass ratios leads to an increasingly more significant filtering effect by the Lorentz force and also to more complicated and less intuitive flow patterns (e.g., see Fig. 5). These effects cause density waves to ‘roll’ into the heliosphere (Hunziker et al. 2022a; Sterken et al. 2022). Measurements and monitoring of these waves may yield information about the structure and the dynamics of the heliospheric magnetic fields (and the dust surface charge) in combination with modeling. The dynamics of ISD in the outer boundaries of the heliosphere is not well known, in particular its time dependence.

The enhancement of gravitational-focusing-induced micron-sized interstellar dust in the solar system, and the filtering due to radiation pressure and the time-and-location-dependent Lorentz force, have been studied extensively by several modelers (see Sect. 4.1).

### Nanodust and Macromolecules

**Nanodust** (≈ 2–30 nm) Although up to 10,000 times more abundant in interstellar space than the submicrometer-sized dust particles, nanodust is almost all filtered out at the heliospheric boundary. Most likely, it will pile up at the outer edges of our solar system, the heliopause (Slavin et al. 2012; Frisch et al. 2022). Interstellar nanodust can be probed in situ with an Interstellar Probe (Brandt et al. 2022; McNutt et al. 2022) outside of the heliosphere; see also Sect. 5.2, Fig. 10 for an illustration of the ISD size distribution. The biggest difference to the physics of submicrometer-sized dust is the charging mechanism that includes the small particle effect, and stochastic charging (see Sect. 2). Its importance for astrophysics cannot be underestimated, in particular in the range from macromolecules to nanodust sizes (see also Sect. 6.4).

### Filtering Effect of the Heliosphere

The ISD gets filtered in the solar system due to (1) the forces discussed above inside the solar system and, (2) in particular, at the outer boundary regions of the heliosphere (the termination shock, heliosheath and heliopause) where the dust grain charges are higher than in the solar system. Currently, the time-dependence of the ISD filtering in the boundary regions is not understood.

Linde and Gombosi (2000) simulated the filtering of the ISD in the heliosphere boundary regions for the defocusing phase of the solar cycle (1996). Although these authors found a cut-off at $0.1\text{--}0.2$ μm ($10^{-16} - 10^{-17}$ kg), Ulysses and Cassini have measured ISD particles with masses down to about 10^−18^ kg (Krüger et al. 2015; Altobelli et al. 2016). Slavin et al. (2012) show the filtering at the boundary regions for two different configurations, focusing and defocusing, albeit with a static IMF. Kimura and Mann (1999, 2000) studied the filtering at the heliopause as well.

**Fig. 6:**
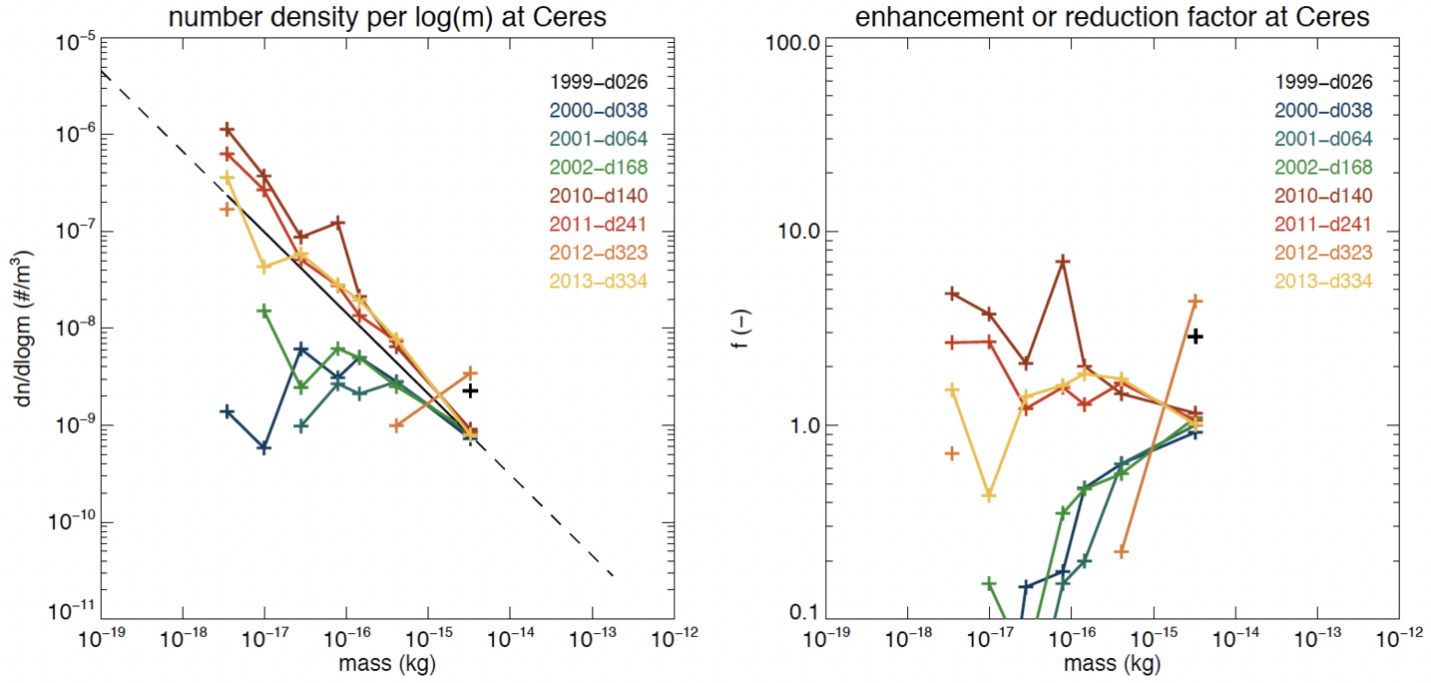
The filtering of ISD (number density per log(m) (left), and filtering factor with respect to the original density in the LIC (right) inside the solar system, not taking the heliosheath filtering into account, over the solar cycle. The MRN size distribution (Mathis et al. 1977) (in the left plot) and adapted astronomical silicates (Sterken et al. 2012) for the composition of the ISD were assumed. Credit: Sterken et al. (2013), p. 11, reproduced with permission $\copyright $ ESO

Filtering inside the solar system plays a major role as well, in particular for the larger particles still subject to the Lorentz force that make it through the heliospheric boundaries and are measured by spacecraft. Trajectory simulations give a clear picture of how the size distribution is altered with the solar cycle by the Lorentz forces closer to the Sun (Sterken et al. 2013). Assuming a power-law for the ISD size distribution in the LIC, such simulations can be used together with spacecraft data (*if* sufficient data exist) to gain more insight in the filtering at the boundary regions. Landgraf et al. (2000), Kimura et al. (2003), and Sterken et al. (2015) studied the filtering of ISD inside the solar system for the Ulysses mission, first assuming astronomical silicates and the MRN size distribution (Mathis et al. 1977) for ISD in the LIC, and later also studying the influence of various dust compositions on the filtered mass distributions. Sterken et al. (2013) reported on the simulated filtering of ISD at different locations in the solar system (the asteroid belt, Jupiter, and Saturn) assuming astronomical silicates adapted to the maximum of the $\beta $-curve derived from Ulysses data by Landgraf et al. (1999). Hunziker et al. (2022b) illustrate how this filtering changes with dust composition for the *Destiny+* space mission, and illustrates how the simpler model of filtering in the solar system only, together with sufficient data from ISD monitoring, can reveal information about the time-dependence of the filtering in the heliosphere boundary. Figure 6 from (Sterken et al. 2013) illustrates an example of filtering at the distance of the asteroid belt throughout the solar cycle (not including the heliosheath filtering). This figure also shows that calibration of the IMEX model (Sect. 4) using Ulysses data must be done carefully using a well-selected time-span in the solar cycle that nevertheless contains sufficient Ulysses data for the calibration of the model with the data.

## Modeling of Dust Flows Through and Near the Heliosphere

### Monte Carlo Trajectory Simulation Models

**Fig. 7:**
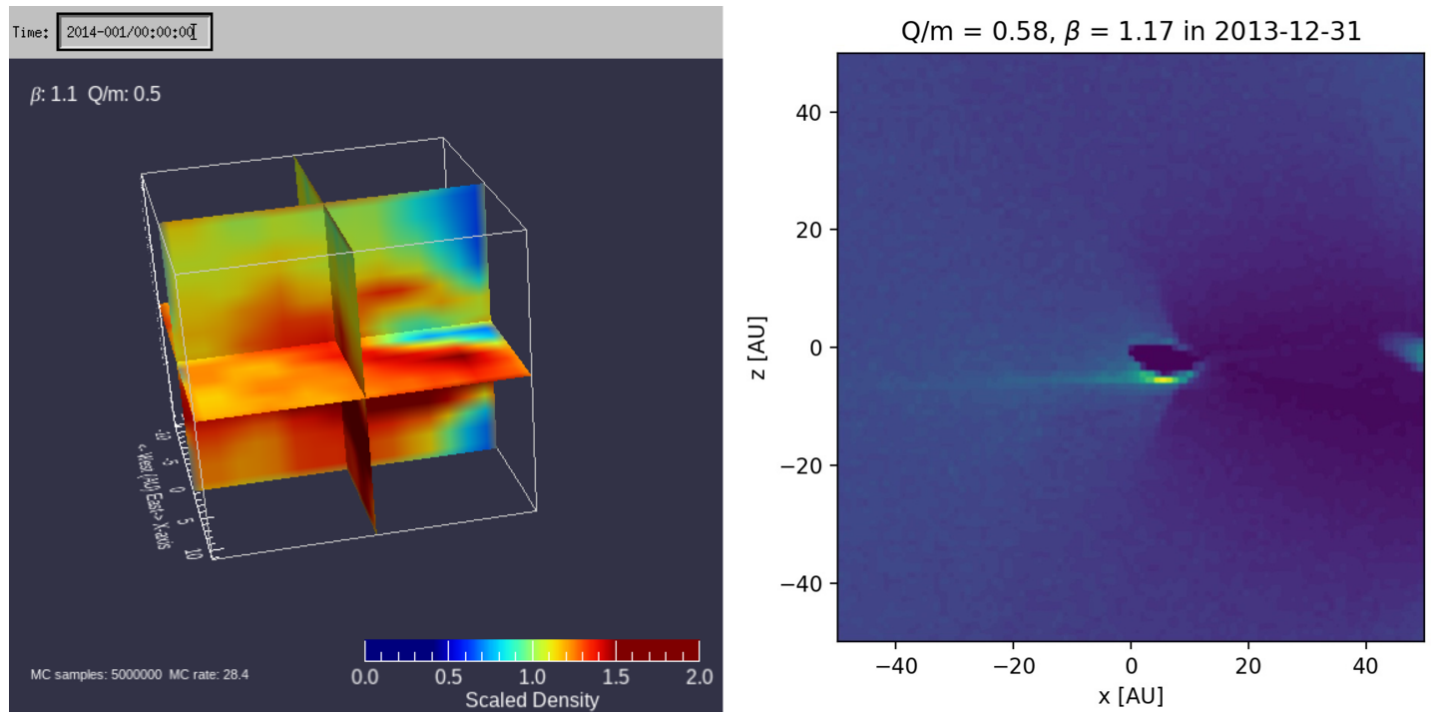
Dust densities in the solar system in a simulation box of 10 AU (left) and of 50 AU (right, IMEX model), as derived from Monte Carlo trajectory simulations (e.g., from trajectories like in Fig. 4)

Monte Carlo simulations^7^ of interstellar dust trajectories in the heliosphere have been made by Landgraf (2000), Sterken et al. (2012), Strub et al. (2011), based on earlier work by Gustafson and Misconi (1979) and Morfill and Grün (1979).^8^ These simulations are based on launching several millions of dust particles from a 2D grid, at 50 to 80 AU upstream from the Sun, with a pre-set flow directionality (typically ca. ecliptic longitude 79^∘^ and latitude −8^∘^ (downstream direction, from Frisch et al. 1999) and speed (e.g., 26 km s^−1^). Dust number densities and averaged velocity vectors are then derived in a smaller box centered around the Sun (e.g., see Fig. 7). The dust particle trajectories were simulated in the solar system under the relevant forces: solar gravitation, solar radiation pressure and Lorentz force. No steady flow of dust is assumed. Until now the heliospheric boundary conditions were not directly implemented in these models, but calibrating them using the dust mass distribution from Ulysses spacecraft data can indirectly reflect the effects of the boundary regions of the heliosphere, albeit at the cost of still having a factor of 2 – 3 difference if the whole 16 years of Ulysses data is used for the calibration^9^(e.g., Sterken et al. 2014; Krüger et al. 2019; Hunziker et al. 2022b). Nevertheless, the currently modeled fluxes, flow directions and mass distributions do not fit the whole time-series of Ulysses data yet (see Sect. 5, and Sterken et al. 2015) and more insight in the effect of the outer regions of the heliosphere is needed. Assuming an MRN-type of distribution (Mathis et al. 1977) and the filtering in the solar system, the spacecraft data and the models can shed light on the filtering function in the heliosheath and its time-variability. Currently, efforts are ongoing to implement the outer boundary regions in such simulations. In previous models, a constant dust surface charge was assumed, which is acceptable inside the solar system where the solar UV and the plasma density both decrease with the square of the distance to the Sun. However, if the heliosheath or regions very close to the Sun are implemented in these models, a variable dust surface charge along the path of the dust particle trajectories needs to be implemented as well. Also the small particle effect needs to be taken into account (see Sect. 2).

### Magnetohydrodynamic Fluid + Kinetic Model

**Fig. 8:**
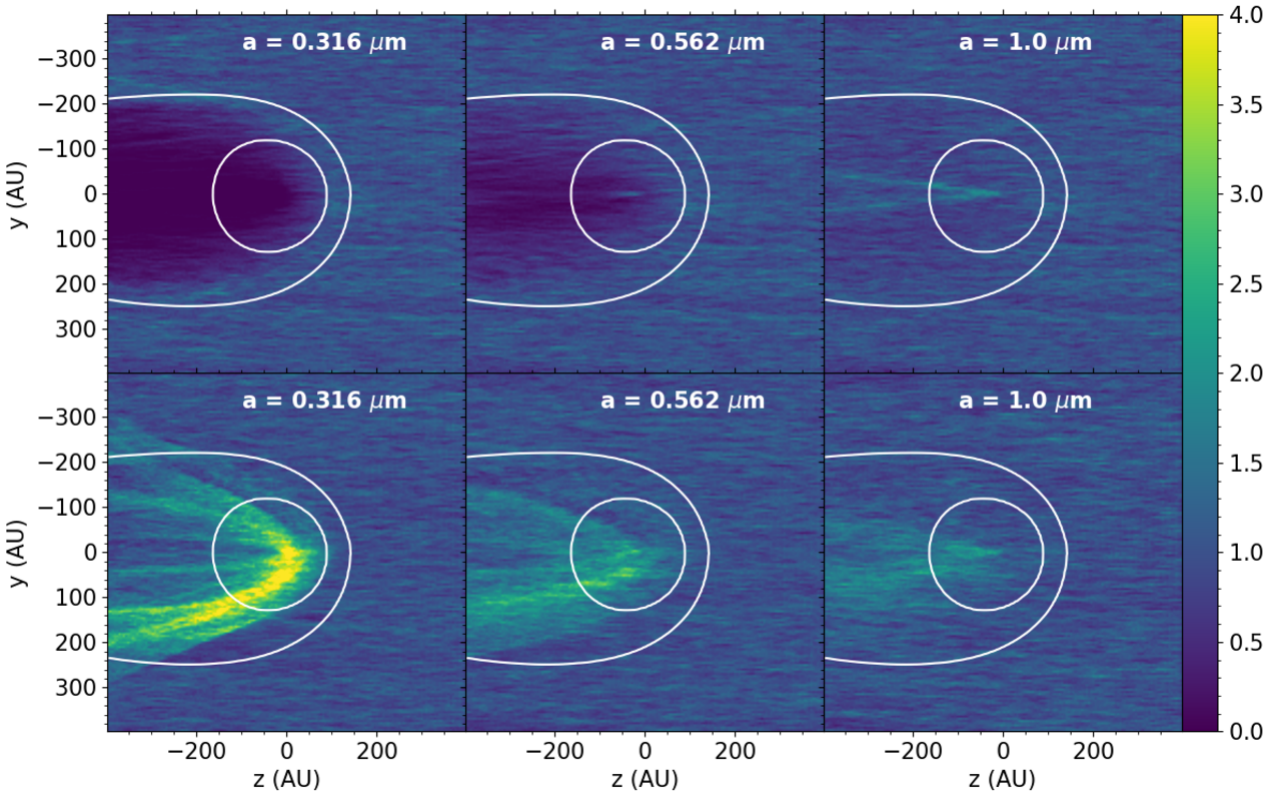
Dust density distributions relative to that in the ISM in a slice parallel to the ecliptic plane (from Slavin et al. 2012, p. 10, but using a different color map). $\copyright $ AAS. Reproduced with permission. The top row is for the defocusing solar wind magnetic polarity and the bottom row is for the focusing polarity (both static). The grain sizes are labeled. The white curves show the locations of the termination shock and the heliopause. The strong dependence on field polarity and grain size are evident

Several studies have investigated the permeability of the heliosheath for ISD (Kimura and Mann 1998, 1999; Linde and Gombosi 2000; Czechowski and Mann 2003b,a; Slavin et al. 2012). Slavin et al. (2012) developed a magnetohydrodynamic fluid and kinetic dust model that was used to investigate the dust density distribution in the whole heliosphere, including the heliosphere boundary regions (Slavin et al. 2012). The model did not include the solar cycle changes of the IMF during the flight path of the ISD (ca. 20 years from the boundary to the solar system), like in the Monte Carlo trajectory models. Instead two different MHD models for the heliosphere were used, one with a focusing polarity of the solar wind magnetic field and one with a defocusing polarity. Those models were calculated by Pogorelov et al. (2008) and Heerikhuisen et al. (2006). The grains were started far upstream of the heliosphere (900 au) in essentially the undisturbed ISM, moving with the gas, though with a small (3 km s^−1^) initial gyrovelocity perpendicular to the assumed interstellar magnetic field. The grain trajectories were then calculated, taking into account the changing magnetic field and plasma conditions with location and the resultant grain charges as they flowed toward, and sometimes into, the heliosphere. An initial 2D grid of starting points was used to allow for the grains to approach from all locations that could lead to a grain entering the heliosphere. The trajectories of the grains were recorded in a 3D grid of locations covering the heliosphere. Since a steady flow of particles was assumed, the time spent by a grain in a cell could be translated into a density of grains in that cell. For each grain size considered, slightly over $10^{6}$ grain trajectories were calculated. In Fig. 8 (from Slavin et al. 2012) we show a slice through the derived dust density distributions for three different grain sizes and for the focusing (bottom) and de-focusing (top) magnetic field polarities. These illustrate the strong dependence of the grain penetration in the heliosphere on grain size and magnetic field polarity.

The advantage of such models is that they correctly treat grains as particles, since grain-grain interactions are not significant on the scale of the heliosphere. As noted above, the models ignore the evolution of the heliosphere with time. More accurate models that include the evolution of the heliosphere are not able to make the steady flow assumption and thus need to follow many more particle trajectories to get the same spatial coverage of the steady flow models. The need for more particle trajectories is challenging computationally and has not been accomplished to date (except for the simplified time-variable models described in Sect. 4.1, which neglect the heliosheath if not using Ulysses data to calibrate these models).

While the Monte Carlo models (Sect. 4.1) accurately describe the filtering in the solar system (out to several tens of AU), the MHD and kinetic model is more accurate for the boundary regions of the heliosphere. A combination of both is needed to unravel the dynamics and filtering of ISD coming in the solar system.

### Lagrangian Fluid Based Models

The flow of ISD particles is considered to be a zero-pressure fluid and the Lagrangian fluid method (Osiptsov 2000) can therefore be used for modeling purposes. These models allow effective study of singular structures of the ISD number density distribution, in particular because such simulations are less computationally expensive than Monte Carlo simulations. Singularities appear due to the Lorentz force on the ISD particles with the alternating magnetic field directions on either side of the heliospheric current sheet (HCS), as demonstrated by Mishchenko et al. (2020). However, these are based on a static phase in the solar cycle during the flight time of the dust in the simulation, similar to the models in Sect. 4.2.

**Fig. 9:**
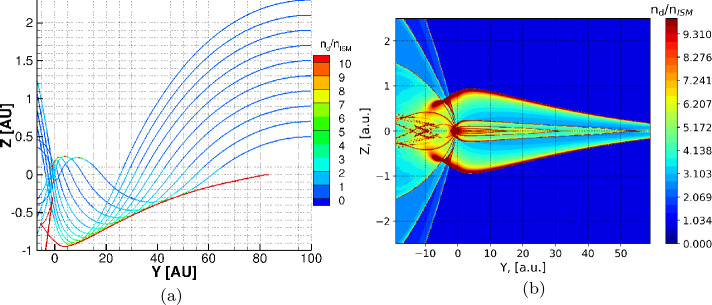
(a) Trajectories of dust particles with a size of 0.37 μm in the $x=0$ plane. The colour corresponds to the number density along the trajectories. (b) The ISD number density for dust particles of the same size but in the cells of the Eulerian grid. Figures 2b and 7 from Mishchenko et al. (2020)

Crossing the HCS, electromagnetic forces on the dust particles reverse causing oscillations of ISD particles around the HCS (see also Czechowski and Mann 2003b). Since the magnitude of the electromagnetic force increases near the Sun (proportional to the azimuthal component of the Parker magnetic field), the amplitude of these oscillations decreases, and, thus, the overall flow of ISD particles is narrowed (see Fig. 9a). The main advantage of the Lagrangian method is that it effectively computates the ISD number density along trajectories, which is inversely proportional to the absolute value of the determinant of the special Jacobian matrix (for more details, see Mishchenko et al. 2020). The trajectories in Fig. 9a have points where the determinant equals zero. Such points taken for different trajectories form a line, a *caustic*, where the number density is infinite. A caustic is the envelope of ISD trajectories, i.e. each finite part of this line is tangent to an infinite number of ISD trajectories, that is the reason the density singularities develop. As shown in Fig. 9b, many caustics and regions of very high density are formed in the vicinity of the HCS. If such features were obtained with more realistic models they could serve as a good starting point in planning future ISD missions.

However, in reality, apart from a time-variable IMF during the travel time of a dust particle, some other factors may prohibit the formation of caustics as well. ISD particles can have non-zero velocity dispersion in the ISM. Singularities disappear even for small values of dispersion (Godenko and Izmodenov 2021) while regular regions of high density remain in the vicinity of the HCS. This occurs because ISD trajectories (launched from a narrow region on the heliosphere boundary) scatter because of the non-zero initial differential velocity of the dust particles. It is known that the dispersion can reach 15%, making it an effect that may be important to consider (apart from the time-variability of the IMF during flight time of the particle).

In summary, a Lagrangian fluid approach is well suited for studying the ISD number density singularities, but the real ambient conditions, in particular the time-variable IMF during the solar cycle while the particles are moving inwards in the heliosphere, likely prevent the appearance of these singularities. Nevertheless, this approach is a useful tool for qualitative analysis.

## In Situ Measurements of ISD Moving in and out of the Heliosphere

### In Situ Measurements by Spacecraft

Interstellar dust has been measured in the solar system with in situ detectors since 1993 (Grün et al. 1993). *Ulysses*, *Cassini*, *Galileo*, *Helios*, and *Wind* have detected ISD in the solar system and some of these data show an imprint of the solar cycle (e.g. *Ulysses*, *Wind*). Landgraf (1998) demonstrated for the first time a solar-cycle dependent flux of ISD in the *Ulysses* data by comparing the data with computer simulations. (Sterken et al. 2015) showed that the 16 years of *Ulysses* data, and in particular the change in flux and flow direction in 2005, could be partially explained by such simulations, but not all the data could be fit. It was postulated that adding the heliospheric interface regions to the simulations could resolve this conundrum. However, this solution would imply that the micron-sized particles are porous, while the particles smaller than about half a micron would be compact, in line with observations from Cassini (compact submicron-sized dust particles, Altobelli et al. 2016) and Stardust (micron-sized porous dust particles Westphal et al. 2014). New dust impact data from the plasma wave instrument are available for the Wind mission (Malaspina et al. 2014; Malaspina and Wilson 2016). This long-term time-series from 1995 until 2020 shows a clear signature of the ISD modulation by the IMF in addition to an interplanetary dust component (Hervig et al. 2022).

A more in-depth review of in situ ISD measurements is given in Sterken et al. (2019) that includes compositional measurements. This chapter mainly describes the modeling and measurements of ISD dynamics and filtering, as it focuses on the dust interaction with the heliosphere and astrospheres.

### The ISD Size Distribution Near the Solar System from in Situ and Astronomical Measurements

**Fig. 10:**
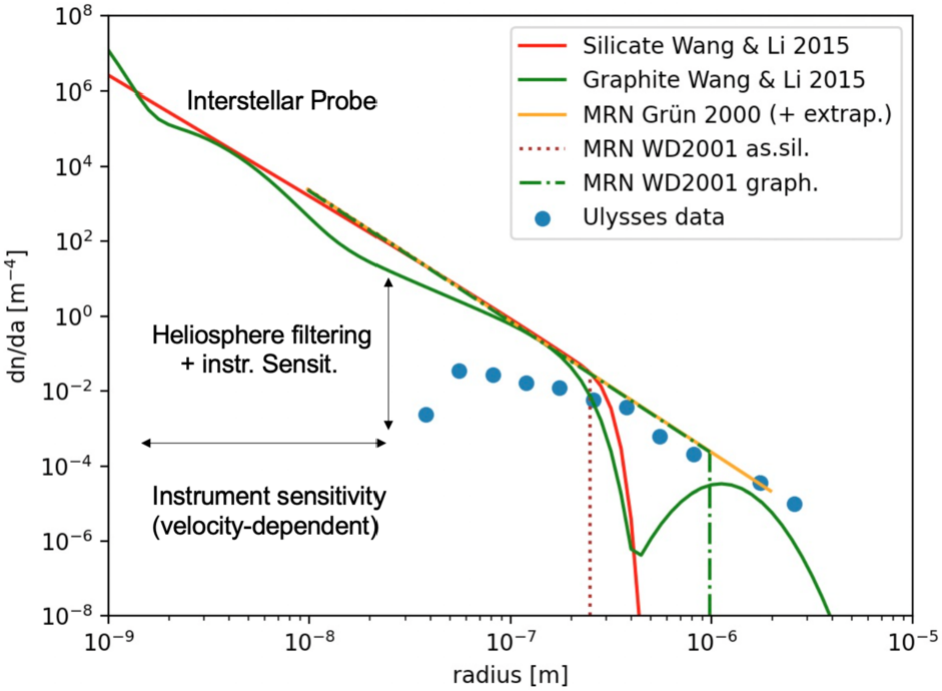
The interstellar dust size distribution from astronomical observations (Wang et al. 2015; Grün and Landgraf 2000; Weingartner and Draine 2001) and 16 years of *Ulysses* data (Krüger et al. 2015). “Interstellar Probe” indicates the regime of the size distribution that can only be measured with an interstellar probe flying beyond the heliopause

While astronomical observations are mostly performed on dust accumulated over long lines-of-sight (typically parsecs to kiloparsecs, see Sect. 6), the in situ dust measurements provide local and ground truth measurements complementary to the astronomical observations. Figure 10 compares the size distributions from current astronomical models based on observations (e.g., Mathis et al. 1977) and from the Ulysses data. The size distribution of interstellar dust near the solar system can be approximated by a power-law distribution with an exponent $\alpha =-3.5$, thus many small dust particles and far fewer micron-sized dust particles exist. The *Ulysses* measurements, however, yielded several micron-sized particles that did not appear in astronomical models which show a maximum size cut-off of 0.3 μm for silicates and 1 μm for graphite (Mathis et al. 1977). Newer models do predict larger grains (e.g. Wang et al. 2015; Gall et al. 2014) but none predict the size distribution observed by Ulysses, which is inconsistent with extinction measurements (Draine 2009b). Smaller particles are underrepresented in the *Ulysses* data in comparison to the size distributions derived from astronomical observations (Landgraf et al. 2000; Sterken et al. 2015), most likely due to the filtering effect of the heliosphere through the Lorentz force, but also instrument sensitivity may play a role. Figure 10 shows several size distributions from models compared with the 16 years of *Ulysses* data that are influenced by the solar radiation pressure, Lorentz force, and instrument sensitivity limitations. An interstellar probe is needed to measure the dust particles in the nanometer size regime. The density of these particles outside the heliopause are expected to be orders of magnitude higher than in the solar system. Summarizing, small particles are missing in the spacecraft data largely due to the filtering of the heliosphere (Sect. 3.4) and the presence in the LIC of large dust grains several micrometers in size is not yet fully understood.

## Remote Sensing Measurements of Interstellar Dust in the Heliosphere Vicinity

In this section, we discuss the available information on the dust associated with the small group of interstellar clouds around the Sun within about 20 pc. This type of dust is (or will be) detected in situ. Measuring ISD properties in nearby clouds and, in particular, in the LIC surrounding the Sun is difficult due to the very low density of the interstellar medium in the vicinity of the Sun. Typical neutral hydrogen columns through clouds at less than ≃20 pc are of the order of 10^18^ to 10^19^ cm^−2^ (e.g., Redfield and Linsky 2008), based on the gas absorption lines in the spectra of nearby stars. As the clouds are ionized to a certain extent, these are lower limits. Nevertheless, the total column densities for both neutral and ionized fractions remain below $5\cdot 10^{19}~\text{cm}^{-2}$ (see Jenkins 2009). As a result, and assuming a classical dust-to-gas ratio, the absorptions and emissions generated by dust grains in the local clouds are extremely small, and it is not easy to measure them or to disentangle their contributions from the contributions of other clouds further away along the same lines of sight. We briefly review the information available from the extinction and polarization of starlight and from the thermal emission from dust.

### Extinction of Stellar Light

**Fig. 11:**
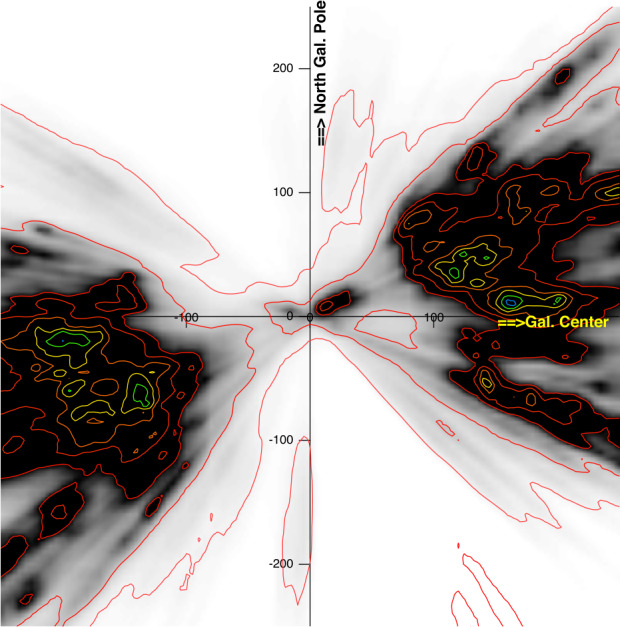
Reconstructed interstellar dust extinction density in a vertical plane containing the Sun and the galactic center direction (the so-called meridian plane). Units are parsecs. The group of clouds in the Sun’s vicinity appears in this new map, despite the low spatial resolution and some “fingers of god” effects, i.e., elongations of the structure in radial directions due to uncertainties of star distances. Note the gap at the exact location of the Sun between two structures, one tenuous, one dense. The figure is derived from the 3D distribution presented in Vergely et al. (2022)

If one uses gas absorptions and typical ratios between gas columns and dust extinctions, extinctions due to local clouds are expected to be of the order of 5 ⋅ 10^−4^ to 10^−2^ mag in the visible. For comparison, state-of-the-art photometric determinations of stellar light extinction for individual targets have uncertainties on the order of 0.2 mag, and even the most precise estimates using both spectroscopy and photometry barely reach 0.05 mag (see, e.g., Vergely et al. 2022). Fortunately, thanks to new, massive spectroscopic and photometric data, and especially thanks to Gaia parallaxes and photometric measurements, it becomes possible to merge data and reconstruct increasingly precisely the 3D distribution of the extinction density, i.e., the extinction light suffers by unit distance, a quantity proportional to the dust grain volume density. Recently, the better spatial resolution allowed mapping of the group of nearby clouds around the Sun. Figure 11 is derived from a 3D extinction density map reconstructed by inversion of about 40 million individual extinction estimates (Vergely et al. 2022). The group of local clouds around the Sun appears distinctly, surrounded by the large volume devoid of dust called the Local Bubble. Similar results were obtained by Leike et al. (2020), based on different data and a different reconstruction technique.

Interestingly, the Sun is located close to (and between) the edges of two neighboring dust concentrations, one denser and more elongated structure in the direction of the inner Galaxy and the second, less dense, in the opposite direction (anti-center). This geometry is in agreement with the local structure deduced from gas absorption data. The Sun is located in the anti-center cloud and moving out of it (Lallement et al. 1993; Witte 2004). This dust-gas geometrical agreement is strong evidence that clouds within 20 pc have properties similar to more distant Galactic clouds. Vergely et al. (2022) used the 3D dust extinction map to integrate from the Sun to nearby white dwarfs located within the Local Bubble (i.e., for which absorption is generated in the group of very nearby clouds and nowhere else) and compared the integrated extinctions with H columns estimated from columns of ionized phosphorus PII and other ions measured in absorption in the white dwarf spectra. They found dust-to-gas ratios fully compatible with classical values. This result is evidence that in-situ measurements of the ISD by a spacecraft outside the heliosphere would probe *normal* interstellar dust.

### Dust Thermal Emission

Column densities of gas can be converted into dust thermal emission using established average relationships (Planck Collaboration et al. 2016). For these numbers, an optical thickness at 353 GHz $\tau $353 of the order of $\simeq3\cdot 10^{-8}$ to $\simeq7\cdot 10^{-7}$ is expected, a signal detectable by the HFI instrument on board the Planck satellite. Such low emissions are measured at high galactic latitudes along directions for which the unique contributions are the very nearby clouds and the low altitude halo (see Fig. 11). Using ratios between optical thickness of $\tau $353 and the gas column derived from full-sky analyses, the above emission level is found to be compatible with the local cloud gas content, an additional argument in favor of a *normality* of the local dust. On the other hand, the dust temperature is estimated to be approximately 22 to 24 K, on the *high* side of temperatures associated with warm gas and low densities. Here again, there does not seem to be any specificity of the nearby ISD. However, Rowan-Robinson and May (2013) modelled the interplanetary dust cloud, including an ISD component as it moves through the solar system, that fit the infrared data from the COBE and IRAS satellites.

### Polarization of Stellar Light

Converting local cloud extinctions or gas columns into polarization fractions, based on observed ratios for lines of sight with higher extinction, gives upper limits of 0.0025% to 0.025% (25 to 250 ppm) for the polarization fraction of nearby stars. Such a weak polarization fraction is of the order of both the instrumental polarization and the intrinsic polarization of active stars (average value of 25 ppm, see Cotton et al. 2017). Its measurement requires instrumentation with exceptional sensitivity and calibration (see, e.g., Skalidis et al. 2018; Cotton et al. 2019; Panopoulou et al. 2019), as well as a careful correction of the sources of contamination. Maximal values of the polarization fraction are reached if the magnetic field is ordered, grains are well aligned, and the magnetic field has a component in the plane of the sky (fields oriented along the line of sight do not produce polarization). This explains why, while polarization fractions and orientations are reliable for stars located beyond dense clouds, e.g., beyond the boundaries of the Local Bubble, the results are more uncertain in the case of nearby targets that probe the local clouds. High-quality measurements of the polarization fraction and polarization angle for the nearest stars were published by Piirola et al. (2020) (see also references therein). The majority of the target stars within 50 pc have a polarization fraction of less than 25 ppm, and there is a high variability of polarization angles across the sky (see their Fig. 3). As Piirola et al. (2020) show, such a lack of homogeneity is opposite to the large scale organized pattern observed for stars between 100 and 500 pc. The exception is two areas that appear to have some continuity of orientation interpreted as a sign of filamentary structure associated with the heliosphere (Frisch et al. 2015, 2022). The local and global patterns and what may influence the nearby magnetic field orientation in the local clouds is unclear. The weakness of the polarization fraction is also a possible sign of weak alignment of the grains.

Fortunately, due to the growing interest in the nearby interstellar medium magnetic configuration, which is important for Cosmic Microwave Background (CMB) polarized foreground removal, there are ongoing efforts to improve polarization measurements. It may expected that such efforts will benefit to the local cloud studies.

### Dust Composition

Sophisticated dust models have been built to explain the bulk of remote multi-wavelength observations of the ISD (see e.g., Fig. 10). However, the actual number of grain types, their size distribution, and their composition still differ among models, and *in situ* data are crucially needed to complement remote sensing.

In this respect, a very interesting finding is the absence of convincing evidence of carbon in the interstellar grains detected *in situ* with the Cassini spacecraft (Altobelli et al. 2016). The Stardust sample return also found no convincing evidence for carbon (these samples were larger than the Cassini dust particles), except for one sample that was partially destroyed upon impact and plausibly may have been carbon-dominated (Westphal et al. 2014). According to a recent model (Jones 2021), carbon is contained in a population of small grains and is mostly absent from the large silicate and graphite grains. The non-detection of carbon may be due to the high charge-to-mass ratio (and/or high $\beta $-values) of the small carbonaceous grains and their subsequent exclusion from the inner heliosphere (see previous sections) or due to exothermic chemical reactions on their pathway into the solar system (Kimura et al. 2020). On the other hand, the remote observations are not inconsistent with a total absence of such grains in the local clouds (Slavin and Frisch 2006), as could happen if the small grains are fully destroyed by shocks or intense radiation. Measurements from an interstellar probe would be of considerable interest in this respect.

The remaining questions also include the role and sites of production and destruction of the many carbonaceous macromolecules that correspond to the intermediate state between grains and gaseous species, and produce the hundreds of irregular absorption bands observed in spectra of objects behind dense clouds, the so-called Diffuse Interstellar Bands (DIBs). Detecting DIBs associated with local clouds would be a step forward in understanding where these particles reside and how they participate in the ISD lifecycle. Unfortunately, DIBs are weak, and up to now their unambiguous detection has been made only in the spectra of distant stars. In the same way that extinction due to the local clouds could be detected by accumulating a large number of individual measurements, it is hoped that an accumulation of spectra of nearby stars could reveal the weak DIBs potentially associated with local clouds. Vast amounts of data recorded with the Radial Velocity Spectrometer (RVS) on board Gaia are being analyzed. The RVS wavelength range contains the 860 nm DIB already detected in many ground-based spectra. Optimistically, results about the local clouds may occur in the near future.

## Measurements of Dust in and Around Astrospheres

Unlike the heliosphere, for other astrospheres no in situ dust measurements can be made. Instead, all knowledge of astrospheric dust must be inferred from remote observations, most notably from the thermal emission of dust. Indeed, the primary channel for detecting and examining astrospheres is the infrared emission of hot dust, yielding surveys and catalogs of astrospheric bow shocks (see, e.g., van Buren et al. 1995; Peri et al. 2015; Kobulnicky et al. 2017, and references therein). The existing classification scheme for observational images of astrospheres by Cox et al. (2012) is based on the thermal emission of dust in the far-infrared.

Studies of astrospheric dust typically begin with detecting the distinctive infrared arc structures and their morphological classification (cf., e.g., the above references for general surveys); more precise investigations into these arcs can often reveal substructures. Katushkina et al. (2018) have compared observations of dust by the *Spitzer Space Telescope* with 3D MHD modeling of the blue supergiant $\kappa$ Cas, finding cirrus-like filaments beyond the arc structure. Similar studies by, e.g., Decin et al. (2012) and Meyer et al. (2021), comparing data from multiple instruments including the *Herschel Space Observatory* with hydrodynamic (HD) modeling of the red supergiant Betelgeuse, have found a linear bar in front of multiple arcs. Gvaramadze et al. (2018) modeled the X-ray binary Vela X-1 and a wedge-like structure in its perturbed environment (cf., e.g., Baalmann et al. 2021) to reproduce the filamentary structure found by spectroscopic dust observations. Investigations of other infrared-bright objects have, under closer scrutiny, revealed astrospheric structures. One example is the well-studied exoplanetary debris system of HR 4796A, which in addition to the ring-like emission of the debris disk, also features a much larger, optically bright exo-ring structure that is suggestive of an astrospheric bow shock (Schneider et al. 2018).

In order to generate a (magneto-)hydrodynamic ((M)HD) bow shock, the relative speed between the star and its environment, which generally is either the ISM at rest or an oncoming stream (Povich et al. 2008), must be supersonic and super-Alfvénic (e.g., Herbst et al. 2022). The domain between this bow shock and the astropause, referred to as the outer astrosheath, the VLISM, or the bow shock shell (e.g., Kleimann et al. 2022), features a high density of gas or plasma and, in most cases, of dust. The emission from this domain is the origin of the observable arc-like structure, which is generally referred to as the observed bow shock. van Marle et al. (2011) found with multifluid HD simulations that the dust’s grain size considerably affects its location within the astrosphere (cf. Slavin et al. 2012, for simulations of the heliosphere region between the bow shock/wave and heliopause); the HD bow shock can lead to multiple shell-like features or a thicker continuous arc of dust emission in observational images.

According to Henney and Arthur (2019a,c), the interaction regions of stars with their respective environments can be divided into four regimes: wind-supported bow shocks (WBSs), radiation-supported bow shocks (RBS), potential dust waves (DWs), and radiation-supported bow waves (RBWs). A brief summary of the environments is given below.

**Fig. 12:**
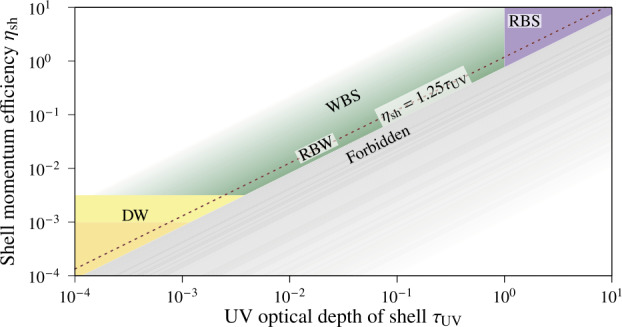
Regimes of interaction regions in the $\eta _{\mathrm{sh}}$-$\tau _{\mathrm{UV}}$-diagram: wind-supported bow shocks (WBSs, top left corner, shaded in green), radiation-supported bow shocks (RBSs, top right corner, shaded in purple), potential dust waves (DWs, bottom left corner, shaded in yellow), and radiation-supported bow waves (RBWs, on the dotted line). The dotted red diagonal line marks $\eta _{\mathrm{sh}}=1.25\tau _{\mathrm{UV}}$, below which no interactions can occur; the bottom right corner, shaded in grey, is strictly forbidden. For details, see Sect. 7. Reproduced after Henney and Arthur (2019c, Fig. 1)

By introducing the optical depth of the bow shell to UV radiation, $\tau _{\mathrm{UV}}$, which can be estimated by the fraction of the observed infrared luminosity of the bow shell’s dust grains to the bolometric luminosity of the star, and the shell momentum efficiency, $\eta _{\mathrm{sh}}\equiv P_{\mathrm{sh}}/P_{\mathrm{rad}}$, which is the ratio of the thermal and magnetic pressure inside the bow shell, $P_{\mathrm{sh}}$, to the stellar radiation pressure, $P_{\mathrm{rad}}$ (Henney and Arthur 2019c, Sect. 2), these four regimes can be visualized as distinct domains in a $\eta _{\mathrm{sh}}$-$\tau _{ \mathrm{UV}}$ diagram (see Fig. 12). It is noteworthy that all four regimes lie on or above the diagonal line

4 $$ \eta _{\mathrm{sh}}\approx 1.25\tau _{\mathrm{UV}} \ ; $$

the area below it is forbidden. In three of these regimes, gas and dust are tightly coupled to each other (Henney and Arthur 2019a), whereas in the fourth they are decoupled (Henney and Arthur 2019b).

Henney and Arthur (2019c) found twenty sources with reliable properties in the sample of observed bow shocks by Kobulnicky et al. (2018). Of these twenty, plus three additional sources, nineteen lie within the regime of the WBSs, which are the default regime for star-environment interaction regions. In this regime, the stellar wind’s ram pressure dominates over the stellar radiation pressure; the astrospherical shock structure therefore follows the (M)HD scenario, and the dust is coupled to the gas. In the $\eta _{\mathrm{sh}}$-$\tau _{\mathrm{UV}}$ diagram this regime lies above the diagonal line.

Of the 23 analysed sources, only four are strong candidates for RBWs (cf. Henney and Arthur 2019c, Sect. 3.5). In this regime, the stellar radiation pressure is more significant and can gradually decelerate the dust. Because gas and dust are still tightly coupled, there is no bow shock but instead a bow wave. In the $\eta _{\mathrm{sh}}$-$\tau _{\mathrm{UV}}$ diagram this regime lies on the diagonal line for $\eta _{\mathrm{sh}}\sim \tau _{\mathrm{UV}}<1$.

If the stellar radiation pressure is stronger still, dominating over the stellar wind’s ram pressure, a RBS is formed. Because gas and dust are tightly coupled, this regime is similar to the WBS; the stellar radiation pressure has taken the place of the stellar wind’s ram pressure. In the $\eta _{\mathrm{sh}}$-$\tau _{\mathrm{UV}}$ diagram this regime lies on the diagonal line for $\eta _{\mathrm{sh}}\sim \tau _{\mathrm{UV}}>1$. No candidate for this regime was found among the 23 analyzed sources.

In the fourth regime, gas and dust are only weakly coupled, forming a DW at further distance from the star compared to the WBS-like gas shock structure (cf. Henney and Arthur 2019b). In the $\eta _{\mathrm{sh}}$-$\tau _{ \mathrm{UV}}$ diagram this regime lies on the diagonal line for $\eta _{\mathrm{sh}}\in \left [3000^{-1}, 1000^{-1}\right ]$; however, DWs additionally require a high relative speed between the star and its environment as well as a discrete range of environmental number densities. No candidate for this regime was found among the analyzed sources either.

## Dust in the Heliosphere, and Its Importance for Future Research

In situ and remote measurements and observations of interstellar (and interplanetary) dust have become of increasing interest for several different reasons. As explained in this chapter, in situ measurements (e.g. Ulysses, Cassini and Stardust) have uncovered discrepancies in the size distribution and composition of interstellar dust when compared to earlier astronomical observations of the dust in the ISM. These discrepancies – and the interaction of ISD with the heliosphere magnetic fields and plasma – raises a number of interdisciplinary science questions to be resolved, of which many relate to the physics, the structure and the dynamics of the heliosphere. A few of these questions are: how does the heliosphere boundary interact with and alter the size distribution, and compositions of the dust grains that are measured in situ in the solar system and how does this depend on time? What is the grain size distribution, composition and homogeneity in the VLISM? How does the ISD-heliosphere interaction play a role in the physics and in the pressure balance of the heliosphere, and can it affect the size and shape of its boundary regions? How does the dust affect pickup ion generation and distributions in the heliosphere? Direct measurements of dust inside and outside of the heliosphere would give us insight into the dust properties in the VLISM and its role in the physics and the shaping of the dynamic heliosphere. In situ measurements of the dust surface charge, dust speed and flow direction at different positions in the solar system and different times in the solar cycle, and advanced computer modeling could be used to indirectly gain insight into space environment-dependent dust charging processes and the solar-cycle variability of the heliosphere structures and size. These are combined efforts of dust, plasma and heliosphere sciences and would require extensive and specialized in situ dust measurements and simulation efforts from different but adjacent fields.

An extended overview of the synergies between dust and heliosphere science and the current open questions is presented in Sterken et al. (2022).

## Footnotes

1 Two exceptions exist: (1) the grain potential may fluctuate with respect to the equilibrium potential for very small grains or grains in a very tenuous plasma, i.e. when charging currents are no longer continuous because of the quantified nature of electric charge, and (2) under dusty plasma conditions where the amount of grain charges could be greatly reduced because of mutual shielding between charged dust grains located in each other’s vicinity.

2 The minimum thermodynamic work needed to remove an electron from the dust particle.

3 Heating by pickup ions is taken into account, which was not yet the case in Kimura and Mann (1998).

4 Approximating the local ISD population as $\sim 0.2~\upmu \text{m}$ spheres with $n_{d}\approx 10^{-13}~\mbox{cm}^{3}$.

5 The dust particles absorb light, and re-emit infrared radiation in a non-isotropical way, due to the rotation of the dust particles.

6 The IDP dynamics are described in Koschny et al. (2019).

7 Trajectory simulations were run for a large number of starting conditions (initial coordinates and time). Their number density and velocities were then averaged out per voxel in a volume surrounding the solar system, allowing comparison with spacecraft data.

8 The model by Strub et al. (2011) is also called the *Interplanetary Micrometeoroid Environment for EXploration (IMEX) model.*

9 A good trade-off between amount of particles per particle size bin and time-span during the solar cycle has to be made for this calibration.
